# Petrophysical characterization of high-rank coal by nuclear magnetic resonance: a case study of the Baijiao coal reservoir, SW China

**DOI:** 10.1098/rsos.181411

**Published:** 2018-12-12

**Authors:** Dongming Zhang, Yapei Chu, Shujian Li, Yushun Yang, Xin Bai, Chen Ye, Decai Wen

**Affiliations:** 1 State Key Laboratory of Coal Mine Disaster Dynamic and Control, Chongqing University, Chongqing 400044, People's Republic of China; 2 College of Resources and Environmental Science, Chongqing University, Chongqing 400044, People's Republic of China; 3 Sichuan Coal Group Furong Company, Sichuan 64402, People's Republic of China

**Keywords:** coal reservoir, porosity, pore structure, nuclear magnetic resonance

## Abstract

To better apply nuclear magnetic resonance (NMR) to evaluate the petrophysical characterization of high-rank coal, six anthracite samples from the Baijiao coal reservoir were measured by NMR. The porosity, *T*_2_ cutoff value, permeability and pore type were analysed using the transverse relaxation time (*T*_2_) spectrum before and after centrifugation. The results show that the *T*_2_ spectrum of water-saturated anthracite can be divided into a discontinuous and continuous trimodal distribution. According to the connectivity among pores, three *T*_2_ spectrum peaks were identified at the relaxation times of 0.01–1.7 ms, 1.7–65 ms and greater than 65 ms, which correspond to the micropores (less than 100 nm), mesopores (100–1000 nm) and macropores (greater than 1000 nm), respectively. Based on the *T*_2_ cutoff value, we divided the *T*_2_ spectrum into two parts: bound fluid and free fluid. By comparing two classic permeability models, we proposed a permeability model to calculate the permeability of anthracite. This result demonstrates that NMR has great significance to the exploration of coal reservoirs and to the understanding of the development of coalbed methane.

## 1. Introduction

Coalbed methane (CBM) is an efficient, clean and high-quality unconventional natural gas with enormous reserves around the world. The importance of CBM for energy has been increasingly recognized, and at present, the United States, Canada, Australia and other countries have achieved large-scale commercial development [1–3]. In China, due to the influence of special geological conditions, the coal reservoirs generally have low porosity, low permeability and high heterogeneity [4]. The quality of a coal reservoir has a significant impact on the accumulation, migration and recovery of CBM. As an important index for evaluating a coal reservoir, the pore structure of coal has attracted the attention of many experts and scholars.

Previous researchers have classified the pore size of coal according to different research purposes; the two most representative are the pore classification scheme developed by the International Federation of Applied Chemistry (micropores: less than 2 nm, mesopores: 2–50 nm, and macropores: greater than 50 nm) [5] and Hodot (micropores: less than 10 nm, transition pores: 10 nm–100 nm, mesopores: 100 nm–1000 nm, and macropores: greater than 1000 nm) [6]. CBM exists in the coal seam primarily in three states: free gas in porous matrix, free gas in natural fractures and trapped gas adsorbed in the matrix. [7]. There are many methods to quantitatively and qualitatively characterize the pore structure of coal. For example, optical microscope and scanning electron microscopy (SEM) observations can characterize the pore structure and morphology of coal [8,9]; the liquid nitrogen adsorption method can obtain information about specific surface area and pore volume of micropores and mesopores in coal [10]; and mercury intrusion porosimetry (MIP) can obtain the pore structure and pore size distribution of mesopores and macropores [11]. However, the aforementioned methods all have certain limitations; for example, optical microscopy and SEM observations can provide a local picture of coal, but they cannot show the spatial distribution of pores. Moreover, the pore structure would be destroyed, and many micropores and microfractures would be produced during the polishing of the sample. Low-temperature nitrogen adsorption cannot characterize the information about mesopores and macropores within the coal. High-pressure mercury may distort the pore structure of coal and thus affect the accuracy of the experimental results [12].

The nuclear magnetic resonance (NMR) technique is an advanced, fast, nondestructive and convenient method that can reflect the integrity of reservoir properties. NMR has become an indispensable tool for petrophysical characterization [13,14]. Since 1990, NMR has been applied to the evaluation of oil and gas reservoirs, such as clastic rocks and carbonates, and can provide information on porosity, pore size, fracture distribution, permeability, crude oil viscosity, oil saturation, etc. [15–17]. Previous researchers carried out numerous NMR experiments and established the relationship between the NMR permeability and porosity, effective porosity and geometric means of the *T*_2_ distribution. In addition, they established a large number of NMR permeability models. [18–20] Among them, the SDR model and Timur–Cotes model are widely used in conventional oil and gas reservoirs. The Timur–Cotes model uses porosity to calculate NMR permeability, while the SDR model uses the geometric means of the *T*_2_ distribution to calculate NMR permeability. As the coal is a media with low porosity, low permeability and complex pore structure, Yao *et al*. [21–24] applied NMR to evaluate the physical properties of coal reservoirs and established a new NMR quantitative characterization of coal properties: the porosity, pore structure, and movable fluid characteristics obtained by analysing the *T*_2_ spectrum of coal. By comparing his results with the Timur–Coates and SDR permeability models, Yao found that there is a large difference between the permeability of air and the permeability calculated by the two classic models. In addition, he found that the permeability strongly correlates with the effective porosity and thus proposed a movable fluid porosity model (PP models). Li [25] found the coals with different ranks have different *T*_2_ distributions and different amplitude and established the NMR porosity and permeability models for coals with different ranks. However, studies related to the pore structure characteristics and NMR permeability model of high-rank coal are rare, and the SDR and Timur–Cotes permeability models exhibit poor performance for high-rank coal.

In this paper, we investigate the petrophysical characterization of the Baijiao coal reservoir using NMR. The porosity, pore type and pore connectivity were analysed from the *T*_2_ spectrum of high-rank coal. According to the *T*_2_ spectra before and after centrifugation, the *T*_2_ cutoff value and effective porosity were calculated, and a permeability model for high-rank coal was established. The research results are helpful to the application of NMR in coal reservoirs and have great significance for the exploration of coal reservoirs and the understanding of CBM development.

## 2. Experiment set-up

### 2.1. Sample preparation

For this study, a large block of coal was collected from the Baijiao coal reservoir, Sichuan, China. The coal was collected between 500 and 600 m depth below the surface using the channel method, then carefully wrapped in airproof packages and immediately transported to the laboratory for experimental analysis. A total of six samples was taken from the same coal for the experiments. The length of the coal samples is 5 cm, and the diameter is 2.5 cm. The proximate analysis of the coal samples is presented in table 1.

**Table 1.** Proximate analysis and vitrinite reflectance of coal sample.

*M*_ad_ (%)	*A*_ad_ (%)	*V*_ad_ (%)	*F*_cad_ (%)	Ro,max (%)
1.58	23.22	9.7	65.5	2.43

### 2.2. Experimental procedures

**Figure 1.**
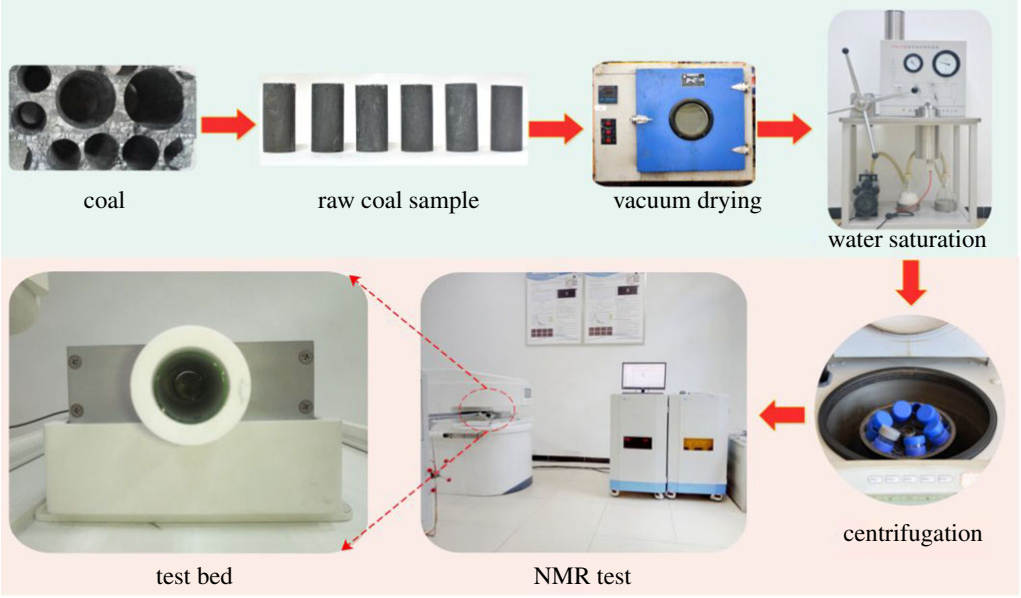
Experimental equipment and procedure.

After the samples were numbered, they were dried with a muffle furnace at 80°C for 12 h until there was no weight change at room temperature (figure 1). Subsequently, these samples were placed into a vacuum water saturation device with a pressure of −0.1 MPa for 12 h until the coal was 100% saturated with water (*S*_w_) (figure 1). The 100% saturated coal samples were measured by NMR spectroscopy to obtain the *T*_2_ spectrum distribution. Then, the coal samples were placed into a centrifuge to be centrifuged until an irreducible water (*S*_ir_) condition was reached; the centrifuge pressure and time were 200 Psi and 90 min, respectively. The irreducible water condition samples were measured again by NMR to obtain another *T*_2_ spectrum distribution.

### 2.3. Nuclear magnetic resonance test

An MR-12-150-I NMR rock corer (Suzhou Niumag Analytical Instrument Co., Ltd., Suzhou, China) was used to analyse the core. The instrument has a primary magnetic field of 0.3 T, a radio frequency (RF) pulse of 1.0–42 MHz and an RF power amplifier of 300 W. The main NMR measurement parameters used in the NMR core analyses were as follows: an RF signal frequency of 32 MHz with the analyses performed with magnets at 32°C; an echo time interval (*T*_E_) of 0.1 ms; a waiting time (*T*_W_) of 1.5 s; the number of scans was 16; the number of echoes was 10 000.

## 3. Results

### 3.1. NMR *T*_2_ distribution of coal

Theoretically, when coal samples with fluid are exposed to a low and uniform magnetic field, the hydrogen atoms within the pore fluid are polarized and generate a magnetic vector [26]. At this time, the samples are exposed to an RF field with a certain frequency, which generates NMR. After removing the RF field, we can obtain an NMR signal whose amplitude decays exponentially with time. Two parameters can be used to measure the attenuation rate of the signal: longitudinal relaxation time (*T*_1_) and transverse relaxation time (*T*_2_). Generally, the transverse relaxation time is used to measure the characteristics of the sample because the measurement method of transverse relaxation time is fast [27].

According to the theory of NMR, the transverse relaxation time (*T*_2_) can be described by the following mathematical equation:

3.1 $$\frac{1}{T_{2}}=\frac{1}{T_{2B}}+\frac{1}{T_{2S}}+\frac{1}{T_{2D}},$$

where *T*_2B_, *T*_2S_ and *T*_2D_ are the bulk relaxation time, surface relaxation time and diffusion relaxation time, respectively. In a low and uniform magnetic field, the diffusion relaxation rate 1/*T*_2D_ approaches zero, and thus the influence of *T*_2D_ on the *T*_2_ distribution can be neglected. Moreover, the bulk relaxation time of the pore fluid *T*_2B_ is greater than *T*_2_, and it contributes little to *T*_2_. Therefore, *T*_2_ is almost entirely determined by surface relaxation time *T*_2S_. Therefore, the equation can be simplified to the following:

3.2 $$\frac{1}{T_{2}}=\rho_{2}\left(\frac{S}{V}\right),$$

where *ρ*_2_ is a constant that represents the relaxation strength (μm/ms), and *S*/*V* is the surface-to-volume ratio that relates to the pore size (cm^−1^).

According to equation (3.2), the distribution of the transverse relaxation time in coal samples reflects the pore sizes [25]: a smaller pore size corresponds to a larger *S*/*V* value and shorter *T*_2_. By contrast, the larger the pore size is in the coal, the lower the value of *S*/*V* and the greater the value of *T*_2_. Fluids in different pores and fractures exhibit different relaxation times; therefore, the amplitude, number, size, area and position of the peaks in the *T*_2_ spectrum distribution can reflect the pore types and pore size distribution. For example, the peak with a larger area reflects the advanced development of pores or fissures and vice versa. Additionally, the continuity of the curve reflects the connectivity between the pores of the coal reservoir. Hence, the pore structure of coal reservoirs can be analysed by the NMR relaxation method.

**Figure 2.**
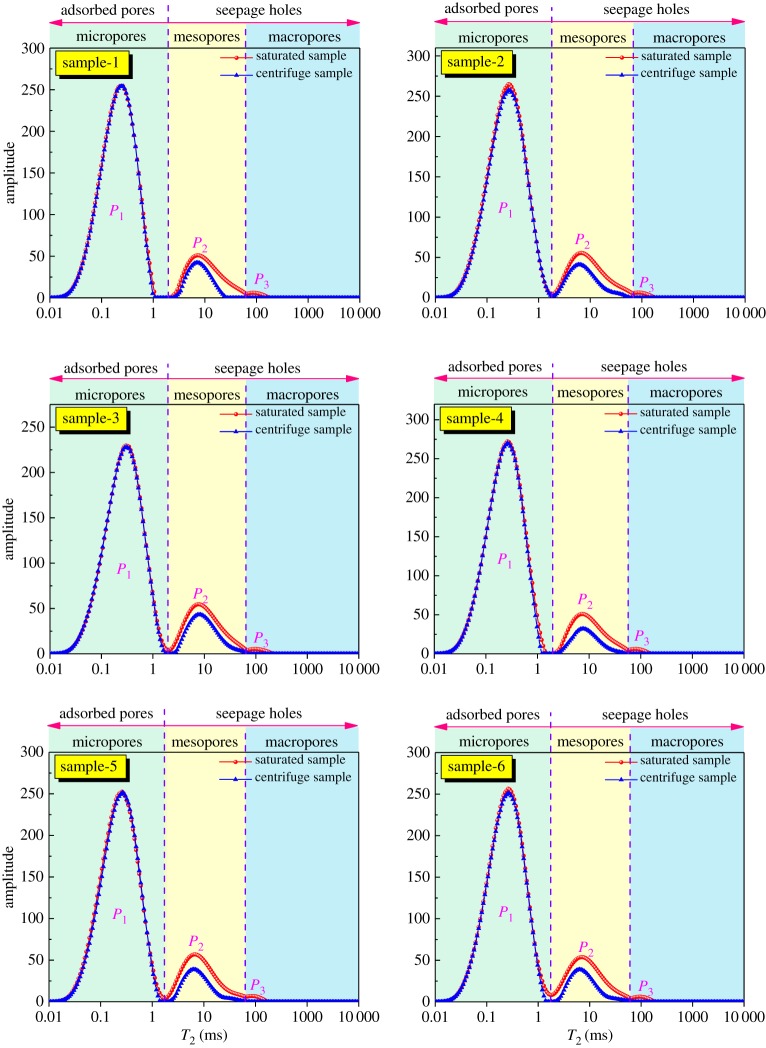
NMR *T*_2_ distribution of coal samples.

In this paper, to simplify the study, we classify the pore structure of coal reservoirs according to the pore classification scheme developed by the Hodot. Based on the pore classification scheme developed by the Hodot [6] and equation (3.2): the *T*_2_ spectrum peak at less than 1.7 ms corresponds to the micropores (pores with diameters smaller than 10 nm) and transition pores (pore with a diameter in the range 10–100 nm) in coal. These pores are adsorbed pores that have a large specific surface area as the main storage space for CBM. The second peak is located between 1.7 ms and 65 ms and corresponds to the mesopores (pore with a diameter in the range 100–1000 nm), while the third peak, which is greater than 65 ms, corresponds to the macropores or microfractures (greater than 1000 nm in diameter). In a study by Cai [28], the mesopores, macropores and microfractures (the second and third peaks in the *T*_2_ spectrum), termed seepage holes, were the main conduits to gas diffusion and permeation in the coal seam. As shown in figure 2, the pores in coal samples are divided into two parts: adsorbed pores and seepage holes.

#### 3.1.1. NMR *T*_2_ distribution for the *S*_w_ condition

The NMR measurement for the *S*_w_ condition was performed for six coal samples to obtain the NMR *T*_2_ spectrum. These *T*_2_ spectra for the *S*_w_ condition are shown as red lines in figure 2. Compared with the typical unimodal or bimodal *T*_2_ spectrum of sandstones [29,30], the *T*_2_ spectra of the selected coal samples exhibit trimodal characteristics. As shown in figure 2, the highest peak located in the low *T*_2_ value section reflects that the micropores and transition pores are dominant in the pore structure of the coal samples. The mesopore peak is smaller in amplitude than the micropore peak, suggesting that the mesopores are relatively developed in coal samples. The macropore peak is the smallest in the amplitude of the three peaks, suggesting that the macropores are not well developed. In addition, the integrated area of the micropores peak is much larger than the subpeak, which implies that the pore structure of the selected coal samples is mainly occupied by micropores and transition pores, while the mesopores and macropores are rarely developed. This result reveals that the adsorbed pores are well developed and the seepage holes are comparatively underdeveloped.

To distinguish the connectivity among pores, we divide the trimodal distribution into a discontinuous trimodal distribution (sample 1 and sample 4) and a continuous distribution (all other samples) based on the connection between the micropore peak and mesopore peak at the *S*_w_ condition. The former discontinuous curve indicates that the coal samples have poor continuity between micropores and mesopores. The latter suggests that coal samples have effective connectivity between the micropores and mesopores.

For the selected coal samples, the volume of adsorbed pores is much higher than the seepage holes, indicating that anthracite has a strong adsorption capacity and high gas content. Because of the poor connectivity between adsorbed pores and seepage holes, the anthracite has a strong adsorption capacity and poor flow ability, which is conducive to CBM enrichment but not beneficial to the migration of CBM.

#### 3.1.2. NMR *T*_2_ distribution for the *S*_ir_ condition

NMR measurements were performed again for the irreducible water (*S*_ir_) condition. The *T*_2_ spectra are shown as blue lines in figure 2. Compared with the *T*_2_ spectrum for the water-saturated (*S*_W_) condition, the micropore peak was apparently not changed. The reason for this is that the fluid in closed or small pores is trapped and cannot be drained by centrifugation due to capillary action and viscous forces. The mesopore peak for *S*_ir_ is distinctly smaller than that of *S*_w_, and the macropore peak totally disappeared after centrifugation. The reason for this is that the connectivity of micropores and mesopores is poor, and the fluid is held in closed micropores and cannot be centrifuged. The fluid in mesopores with moderate connectivity can flow out by centrifugation, whereas the fluid in macropores with good connectivity can be completely expelled by centrifugation.

## 4. Discussion

### 4.1. Porosity test result

In coal reservoir characterization, porosity is an important factor for evaluating the permeability of coal seams that affect the enrichment and diffusion of CBM. The low-field NMR *T*_2_ spectrum can be converted into the NMR total porosity, *φ*_N_, of coal for the water-saturated condition, and the results are shown in table 2. Theoretically, the NMR porosity represents the pore volume fractions occupied by bound and free fluid [22]. By definition, the bound fluid corresponds to the fluid that cannot move by centrifugation due to capillary and viscous forces that can be obtained from the *T*_2_ spectrum for irreducible water condition, while the free fluid can be expelled by centrifugation. Thus, the NMR porosity includes the residual porosity that corresponds to the fraction of bound fluid, *φ*_NB_, and the effective porosity corresponds to the fraction of free fluid, *φ*_NF_. The effective porosity of coal can be acquired by subtracting the residual porosity from the total porosity. Then, *φ*_NB_ and *φ*_NF_ can be calculated by the following equations:

4.1 $$\varphi_{\mathrm{NB}}=\varphi_{N}\times \frac{\mathrm{BVI}}{\mathrm{BVI}+\mathrm{FFI}}$$

and

**Figure 3.**
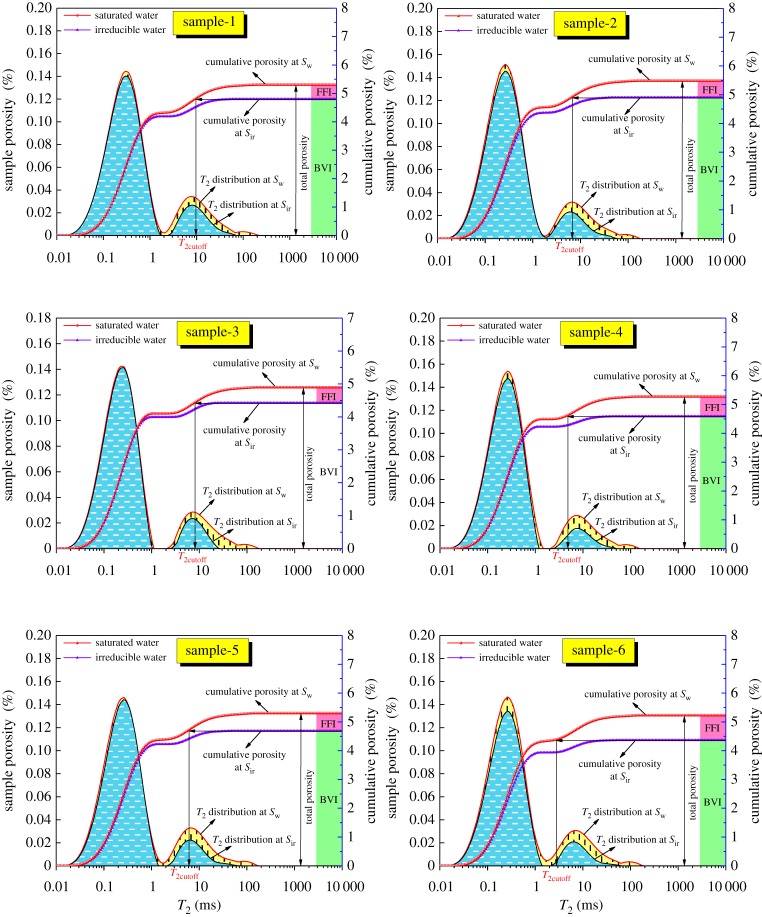
The *T*_2cutoff_ value for coal samples with NMR measurements for the 100% water-saturated condition (*S*_w_) and irreducible water condition (*S*_ir_).

4.2 $$\;\varphi_{\mathrm{NF}}=\varphi_{N}\times \frac{\mathrm{FFI}}{\mathrm{BVI}+\mathrm{FFI}},$$

where *φ*_N_, *φ*_NB_ and *φ*_NF_ are the NMR total porosity, residual porosity and effective porosity, respectively. BVI is the bound fluid index; FFI is the free fluid index. BVI + FFI represents the total fluid that includes bound fluid and free fluid, the BVI and FFI can be calculated according to the cumulative porosity in the *S*_W_ and *S*_ir_ condition shown in figure 3.

**Table 2.** NMR total porosity, residual porosity, effective porosity and *T*_2_ cutoff value of coal sample.

sample	*φ*_N_ (%)	*φ*_NB_ (%)	*φ*_NF_ (%)	BVI(BVI + FFI) (%)	FFI (BVI + FFI) (%)	*T*_2cutoff_
1	5.31	4.80	0.51	90.41	9.59	9.01
2	5.49	4.90	0.59	89.29	10.71	6.37
3	4.90	4.42	0.48	90.24	9.76	7.84
4	5.28	4.59	0.69	86.91	13.09	4.20
5	5.30	4.68	0.62	88.29	11.71	5.54
6	5.23	4.36	0.87	83.34	16.66	2.25

As shown in table 2, the residual porosity ranged between 4.36% and 4.90%, with a mean value of 4.62%. The effective porosity ranged from 0.48% to 0.87% with an average of 0.63%, which is much lower than residual porosity, and the effective porosity of the samples was less than 1%. The proportions of BVI and FFI are obvious in figure 3. There is no direct relationship between effective porosity and total porosity; some samples have a high total porosity, but the effective porosity is low, and vice versa. The effective porosity is determined by the development of macropores and fractures in coals.

### 4.2. *T*_2_ cutoff value

By definition, a condition *T*_2_ cutoff value (*T*_2cutoff_) is the relaxation time boundary that divides the *T*_2_ spectrum for the S_W_ condition into two parts: the bound fluid and the free fluid. The bound fluids in adsorbed pores have a low value (less than *T*_2cutoff_) and the free fluids in seepage holes have a larger value (greater than *T*_2cutoff_). The fraction on the left of *T*_2cutoff_ in the *T*_2_ spectrum corresponds to bound fluid, while the other fraction corresponds to the free fluid. The bound fluid corresponds to the adsorbed pores with poor connectivity that cannot be expelled due to capillary forces, while the free fluid represents the seepage holes that can be drained. The accurate calculation of the *T*_2_ cutoff value is key to distinguishing free fluid and bound fluid as well as the porosity, permeability and pore size distribution; this helps to apply NMR in the evaluation of coal reservoirs.

First, the two *T*_2_ spectra for the water-saturated (*S*_W_) condition and the irreducible water (*S*_ir_) condition must be acquired. Second, the two *T*_2_ spectra are accumulated sequentially to obtain the cumulative porosity curve according to the following method: the maximum cumulative amplitudes of the *T*_2_ spectra for *S*_W_ and *S*_ir_ are equivalent to total porosity and residual porosity, respectively, as shown in figure 3. The difference between the total porosity and residual porosity is the effective porosity. Lastly, a horizontal line from the maximum of the cumulative porosity curve for *S*_ir_ intersects with the cumulative porosity curve for *S*_w_ at one point; then, a vertical line is constructed through the point, and the *T*_2_ value at the intersection that is projected onto the *T*_2_ axis is the *T*_2cutoff_ point.

The calculated *T*_2cutoff_ for the six samples ranges from 4.21 to 9.01 ms with an average of 5.87 ms, as shown in table 2. The greater the *T*_2cutoff_ is, the higher the content of bound fluid in coal, namely, coal with a greater *T*_2cutoff_ has a lower effective porosity. These values are lower than other rocks such as sandstones and limestone [31,32]. The main reason for these differences is that the *T*_2_ cutoff is not only affected by the lithology but also related to pore throat, particle size, texture and measurement parameters.

### 4.3. Permeability

Permeability is an important indication of the capability for CBM migration of coal reservoirs and therefore is a key property for evaluating the production performance of coal reservoirs, and it depends primarily on the connectivity of pores, pore size and effective porosity. Although NMR cannot directly measure permeability, it can be calculated by the porosity, bound fluid index, free fluid index, geometric mean and pore size distribution reflected by the *T*_2_ spectrum. In this study, the two classic permeability models, Schlumberger–Doll research (SDR) and Timur–Cotes (also called free fluid model) were analysed.

#### 4.3.1. SDR model

The SDR model for calculating permeability can be described by the following equation [18,29]:

4.3 $$k_{\mathrm{SDR}}=a{\left(T_{2\mathrm{ga}}^{}\right)}^{n}{\left(T_{2\mathrm{gb}}^{}\right)}^{m},$$

where *k*_SDR_ is the NMR permeability calculated by this equation of the SDR model (10^−3^ µm^2^), *a*, *m* and *n* are constants related to the characteristics rock-coal masses, and $T_{2\mathrm{ga}}^{}$ and $T_{2\mathrm{gb}}^{}$are the geometric means (ms) of the *T*_2_ distribution of coal samples for the water-saturated and irreducible water conditions, respectively.

After a regression analysis, the constants *a*, *m* and *n* can be calculated with an *R*^2^ of 0.736, and the equation can be expressed as the following formula:

4.4 $$k_{\mathrm{SDR}}=0.4468\times {\left(T_{2\mathrm{ga}}^{}\right)}^{-4.938}\times {\left(T_{2\mathrm{gb}}^{}\right)}^{3.895}.$$

#### 4.3.2. Timur–Cotes model

The Timur–Cotes model can be expressed as the following equation [33]:

4.5 $$k_{\mathrm{TC}}=a\varphi^{m}{\left(\frac{\mathrm{FFI}}{\mathrm{BVI}}\right)}^{n},$$

where *a*, *m* and *n* are the fitting parameters, *φ* is the NMR total porosity, and FFI and BVI are the free fluid index and bound fluid index, respectively. After the multiple regression analysis, with a goodness-of-fit of up to *R*^2^ = 0.89, *a*, *m* and *n* are obtained. As a result, the Timur–Cotes model can be written as:

4.6 $$k_{\mathrm{TC}}=1.35\varphi^{0.92}{\left(\frac{\mathrm{FFI}}{\mathrm{BVI}}\right)}^{1.718}.$$

#### 4.3.3. The model proposed in this study

By analysing the above two models, it can be seen that the SDR model relates to the geometric mean of the *T*_2_ distribution, while the Timur–Cotes model is mainly a function of porosity and fluid index. The permeability of coal is mainly constrained by the pore structure, while the anthracite is mainly occupied by adsorbed pores, and the seepage holes are relatively underdeveloped. Most adsorbed pores are closed pores, and the connectivity between adsorbed pores and seepage holes is poor, so they do not contribute to permeability. Thus, the effective porosity is the main factor that affects permeability. A power exponent equation between the air permeability and effective porosity shows a high goodness-of-fit of 0.97, and the model can be determined as:

4.7 $$k_{\mathrm{NF}}=0.5477\varphi_{\mathrm{NF}}^{1.8102},$$

where *k*_NF_ is the estimated NMR permeability determined by the model proposed in this paper.

**Figure 4.**
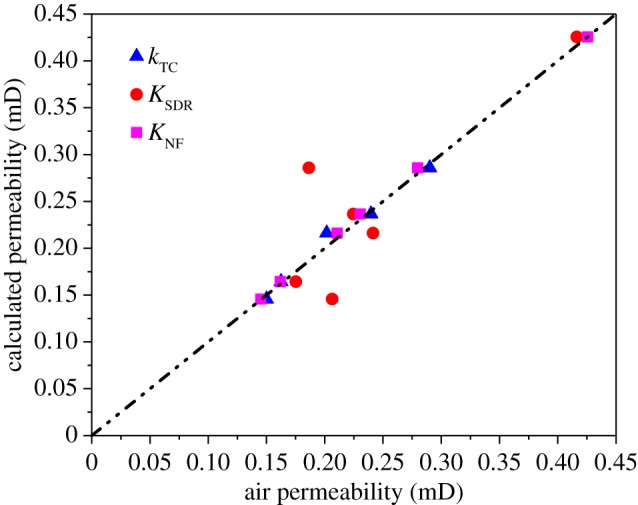
Relationship between the air and NMR permeability.

Figure 4 shows the relationship between NMR permeability that is estimated by the three models and air permeability. All three models can be used to calculate coal permeability, but the model proposed in this paper is more convenient and accurate than the other models because it only requires one parameter, effective porosity, and considers the effect of effective porosity on permeability. Therefore, this model can be used to calculate the permeability, which can meet the demands of research and production.

### 4.4. Pore types

**Figure 5.**
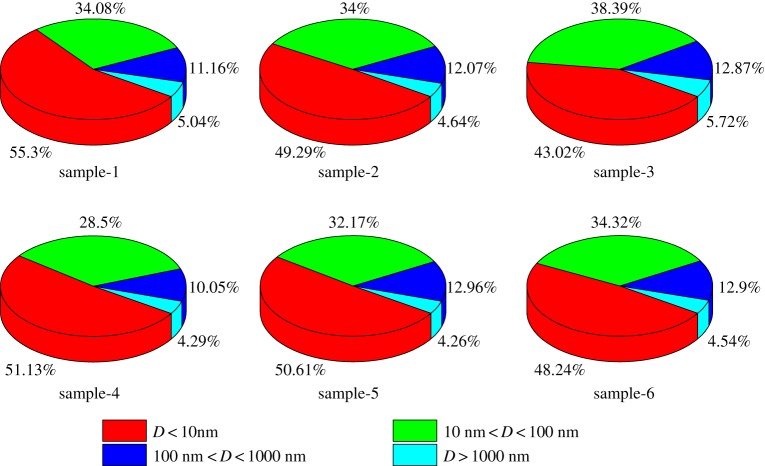
Pore size distribution of six coal samples from NMR measurements.

To further analyse the pore types of anthracite, the *T*_2_ distributions of six coal samples were transformed into pore size distribution according to equation (3.2). As shown in figure 5, in the six coal samples, micropores account for almost half of the proportion, with an average ratio of micropores in the six coal samples of 49.6%; the average ratio of transition pores is 33.58%, the average ratio of mesopores is 12%, and the average ratio of macropores is 4.75%, which indicates that the mesopores and macropores of anthracite gradually closed under the action of pressure and temperature in the process of coalification, resulting in the sharp reduction of flow space. With the continuous change in the molecular structure of coalification, the arrangement of aromatic rings became more ordered, thus, micropores and transition pores of anthracite are extremely well developed, [25] the proportion of adsorbed pores is much larger than that of seepage holes, which implies that the pore structure of anthracite is mainly occupied by adsorbed pores. The anthracite has a strong adsorption capacity and high gas content, which is conducive to the enrichment of CBM. Correspondingly, the mesopores and macropores are relatively underdeveloped, and the seepage holes occupy a small proportion. Therefore, the flow space is underdeveloped, which is not beneficial to the desorption, diffusion and production of CBM.

## 5. Conclusion

In this paper, the petrophysical characterization of six coal samples from the Baijiao coal reservoir was studied using NMR, and the following conclusions can be made:

(1) The NMR *T*_2_ spectrum for the 100% water-saturated condition can be divided into discontinuous and continuous trimodal distributions based on the connectivity among pores. The micropore peak (less than 100 nm) is at 0.01–1.7 ms, the mesopore peak (100–1000 nm) is at 1.7–65 ms and the macropore peak (greater than 1000 nm) is at greater than 65 ms.
(2) The NMR total porosity was measured by using NMR for the 100% water-saturated condition; then, the coal samples for the irreducible water condition were obtained by centrifugation, and the effective porosity of the coal samples was calculated. The effective porosity of coal samples was low, ranging from 0.48% to 0.87% with an average of 0.63%.
(3) The *T*_2_ cutoff value of anthracite ranges from 4.21 to 9.01 ms with an average of 5.87 ms. The greater the value of *T*_2cutoff_, the higher the content of bound fluid in the coal; coal with a greater *T*_2cutoff_ has a lower effective porosity.
(4) The pore types of anthracite can be obtained by converting the *T*_2_ distribution of the coal sample into a pore size distribution. The micropores and transition pores were the major components of the pore structure of anthracite. This result shows that the anthracite was mainly occupied by adsorbed pores, which is conducive to the enrichment of CBM. The mesopores and macropores are relatively underdeveloped, and the flow space is underdeveloped, which is not beneficial to the desorption, diffusion and production of CBM.

## Supplementary Material

T2 curve of coal samples

## Data accessibility

We include all the experimental data in the electronic supplementary material, which are available at Dryad Digital Repository: http://dx.doi.org/10.5061/dryad.4r34459 [34].
